# Increased stromal PFKFB3-mediated glycolysis in inflammatory bowel disease contributes to intestinal inflammation

**DOI:** 10.3389/fimmu.2022.966067

**Published:** 2022-11-02

**Authors:** Zhou Zhou, Leonie G. Plug, Thiago A. Patente, Eveline S. M. de Jonge-Muller, Amir Abou Elmagd, Andrea E. van der Meulen-de Jong, Bart Everts, Marieke C. Barnhoorn, Lukas J. A. C. Hawinkels

**Affiliations:** ^1^ Department of Gastroenterology and Hepatology, Leiden University Medical Center, Leiden, Netherlands; ^2^ Department of Parasitology, Leiden University Medical Center, Leiden, Netherlands

**Keywords:** inflammatory bowel disease, PFKFB3, stromal cells, glycolysis, fibroblast

## Abstract

Inflammatory bowel disease (IBD) is a chronic relapsing inflammation of the intestinal tract with currently not well-understood pathogenesis. In addition to the involvement of immune cells, increasing studies show an important role for fibroblasts in the pathogenesis of IBD. Previous work showed that glycolysis is the preferred energy source for fibroblasts in fibrotic diseases. 6-phosphofructo-2-kinase/fructose-2, 6-bisphosphatase 3 (PFKFB3) is a key kinase supporting glycolysis. Increased expression of PFKFB3 in several cancers and inflammatory diseases has been previously reported, but the metabolic status of fibroblasts and the role of PFKFB3 in patients with IBD are currently unknown. Therefore, in this study, we evaluated the role of glycolysis and PFKFB3 expression in IBD. Single-sample gene set enrichment analysis (ssGSEA) revealed that glycolysis was significantly higher in IBD intestinal samples, compared to healthy controls, which was confirmed in the validation cohorts of IBD patients. Single-cell sequencing data indicated that PFKFB3 expression was higher in IBD-derived stromal cells. *In vitro*, PFKFB3 expression in IBD-derived fibroblasts was increased after the stimulation with pro-inflammatory cytokines. Using seahorse real-time cell metabolic analysis, inflamed fibroblasts were shown to have a higher extracellular acidification rate and a lower oxygen consumption rate, which could be reversed by inhibition of JAK/STAT pathway. Furthermore, increased expression of pro-inflammatory cytokines and chemokines in fibroblasts could be reverted by PFK15, a specific inhibitor of PFKFB3. *In vivo* experiments showed that PFK15 reduced the severity of dextran sulfate sodium (DSS)- and Tcell transfer induced colitis, which was accompanied by a reduction in immune cell infiltration in the intestines. These findings suggest that increased stromal PFKFB3 expression contributes to inflammation and the pathological function of fibroblasts in IBD. Inhibition of PFKFB3 suppressed their inflammatory characteristics.

## Introduction

Over the past decades, an increasing incidence of inflammatory bowel disease (IBD) has been reported (1). Despite extensive research efforts, the exact etiology and pathogenesis of IBD remain to be determined. IBD is characterized by aberrant and continuing immune response in the intestinal tract which results in chronic relapsing inflammation (2). It comprises ulcerative colitis (UC) and Crohn’s disease (CD). Patients with IBD regularly suffer from abdominal pain, diarrhea, rectal bleeding, and weight loss (3). At the cellular level, immune cells, involved in initiating and maintaining inflammation, are mostly studied (4–6). Recently, the importance of stromal cells, which are defined as non-hematopoietic, non-epithelial, and non-endothelial cells, has been highlighted in the pathogenesis of IBD (7, 8). Stromal cells do not only provide structure to the intestines but also regulate epithelial and immune cell homeostasis (9). In inflammatory conditions, stromal cells participate in wound healing, extracellular matrix remodeling, and immunoregulation (10, 11). The most abundant stromal cells are fibroblasts. Fibroblasts derived from IBD patients were reported to proliferate faster and secret more collagen compared to healthy control (12). This means that fibroblasts involve in epithelial layer repair and also the fibrosis of IBD patients. Furthermore, myofibroblasts express less programmed death-ligand 1 (PD-L1) in inflamed CD, which contributes to the persistence of a T helper type 1 (Th1) inflammatory milieu (13).

Glucose is the main source of energy for cell metabolism with glycolysis being one of the most important metabolic pathways (14). Activated immune cells have an increased glycolytic rate to meet the high energetic and biosynthetic demand (15, 16). Thus, targeting glycolysis in immune cells would shift the metabolic flux, resulting in the alleviation of pathological inflammation (17, 18). Studies in fibrotic diseases revealed that glycolysis is the preferred energy source for fibroblasts as well (19, 20). Inhibition of glycolysis decreases extracellular matrix (ECM) production of fibroblasts, thereby decreasing fibrosis (21). In rheumatoid arthritis, fibroblast-like synoviocytes prefer glycolysis, accompanied by a higher expression of glucose transporter 1 (GLUT1) and 6-phosphofructo-2-kinase/fructose-2, 6-bisphosphatase 3 (PFKFB3) (22, 23). In the glycolysis pathway, PFKFB3 is a key regulator of glycolytic activity (24). It encodes 6-phosphofructo-2-kinase/fructose-2,6-biphosphatase 3 enzyme which catalyzes the synthesis and degradation of fructose-2,6-bisphosphate (Fru-2,6-P_2_). Fru-2,6-P_2_ is a potent activator for phosphofructokinase-1 (PFK1) which is a rate-limiting step of glycolysis (25). PFKFB3 inhibitors have been reported as potential anticancer agents (26, 27). In addition, it was also reported that inhibition of PFKFB3 improved the status of some benign diseases, such as rheumatoid arthritis and atherosclerosis (23, 28).

It has been shown that PFKFB3 can have direct involvement in inflammation and various (immune-related) diseases; however, until now, the metabolic status of fibroblasts and the role of PFKFB3 in IBD has not been investigated. Hence, our research aims to elucidate the role of stromal PFKFB3 in the pathogenesis of IBD. In this work, we studied PFKFB3 expression in human IBD tissue samples, showed its role and regulation in primary IBD-derived stromal cells, and investigated its blocking as a therapeutic potential in two IBD mouse models. These data show a previously unknown role for stromal PFKFB3 expression in IBD pathogenesis.

## Materials and methods

### Microarray data collection and single-sample gene set enrichment analysis

mRNA expression profiles of human IBD samples, including GSE16879 (29), GSE186582 (30), GSE59071 (31), GSE73661 (32), GSE75214 (33), GSE87466 (34), GSE112366 (35), and GSE179285 (36), were downloaded from Gene Expression Omnibus (GEO) database (http://www.ncbi.nlm.nih.gov/geo/). All information concerning the platform and the sample details are presented in **Supplementary Table 1**.

To evaluate the glycolysis levels, the GSVA package in R and “ssgsea” method was used. The gene set for glycolysis was obtained from the reactome database (https://reactome.org/). Here, we used all the datasets mentioned above to calculate the normalized enrichment score (NES) to represent the relative amount of glycolysis level of control, UC (including active and inactive), and CD (including non-inflamed and inflamed, active and inactive) samples.

To investigate the correlation between *PFKFB3* and immune cell infiltration, we first obtained the marker gene sets for infiltrating immune cell types from the studies of Bindea et al. (37). Both innate immune cells (e.g., dendritic cells (DCs), eosinophils, mast cells, macrophages, natural killer cells, neutrophils) and adaptive immune cells (e.g., B cells, T cells, T helper cells, CD8+ T cells, Treg cells, and cytotoxic cells) were included. The NES calculated by ssGSEA was used to represent the relative amount of each infiltrating immune cell in intestinal samples. Spearman’s correlation analysis R was performed on 8 datasets.

### Single-cell RNA sequencing analysis

To explore the *PFKFB3* expression pattern in stromal cells, the public data GSE114374 (38), consisting of colon samples from two healthy and two UC patients, and GSE134809 (39), including 11 paired resection ileal samples from the inflamed and uninflamed areas of CD for the single-cell RNA sequencing (scRNA-seq) were retrieved from the GEO database. We used the cell gene expression matrices for further analysis by using the R package Seurat version 4.0.3 (40). After filtering low-quality cells (default setting, min.cells = 3, min.features = 200), reciprocal principal component analysis (PCA) was used for data integration, nearest-neighbor graphs using the top 30 dimensions of the PCA reduction were calculated and the clustering analysis was applied with a resolution of 0.2 using UMAP. The “FindMarkers” function (default setting, min.pct = 0.25, log_2_Fold change threshold = 0.25) was applied to identify the markers that defined each cluster, which automatically, *via* differential expression analysis, compared a cluster against all others. Cluster annotations were based on canonical marker genes. In GSE114374, from Kinchen et al. (39), we used exclusion markers including *PECAM1* (endothelial cells), *S100B* (glial cells), *RGS5* (pericytes), and *SDC1* (plasma cells) to define stromal cells. In GSE134809, markers for fibroblasts (*CCL13*, *CCL8*) and activated fibroblasts (*CXCL8*, *CXCL3*, *CXCL1*, and *CXCL6*) were used to identify stromal cells (38).

### Human samples

All samples from patients with IBD (both UC and CD) were available from the established biobank of the Department of Gastroenterology and Hepatology of the LUMC according to the Code of Conduct for Responsible Use of human tissues and after written consent for biobank was obtained. Samples were fixed in 4% paraformaldehyde followed by paraffin embedding or directly snap frozen and stored at -80°C.

### Fibroblast isolation and culture

Primary intestinal fibroblasts were isolated from endoscopic biopsies from patients with IBD. Inflammation status was endoscopically assessed by the endoscopist. To isolate fibroblasts, the tissue was washed with HBSS (Gibco) and treated with a 3:1 collagenase (Gibco/Thermo Fisher Scientific, Leiden, The Netherlands) and dispase II (Roche, Basel, Switzerland) mix. The mixture was incubated in the water bath at 37°C for 1.5 hours and vortexed every 30 minutes. The single cell suspension was collected and cultured in Dulbecco’s modified eagles’s medium (DMEM)/F12/Glutamax (Thermo Fisher Scientific) with 10% fetal calf serum (FCS), 100 IU/ml penicillin/streptomycin (P/S), 2.5 ug/ml fungizone and 50 ug/ml gentamicin (all Thermo Fisher Scientific) at 37°C, 5% CO_2_ until outgrowth of fibroblast-like cells was observed. The fibroblast-like cells were then characterized by quantitative polymerase chain reaction (qPCR) and used in future experiments when there was expression of fibroblast marker *ACTA2* (alpha smooth muscle actin, *α-SMA*) and no expression of *PTPRC* (*CD45*), *KRT20*, and *PECAM1* (*CD31)* to exclude cells of immune, epithelial, and endothelial origin, respectively (**Figure S1**). Fibroblasts were used in the following experiments between passages 3 and 10.

To assess the regulation of PFKFB3 expression under inflammatory conditions, cells were seeded in 6-well plates (Coaster Corning Incorporated) at 100,000 cells per well. After 24 hours, they were treated with PFK15 (1µM; Selleck Chemicals, Houston, USA), a selective inhibitor of PFKFB3, and/or tumor necrosis factor alpha (TNF-α, 10ng/ml; Peprotech, Rocky Hill, NJ, USA), and/or a cytokine mix, including interleukin (IL)-17A (50ng/ml; Peprotech, Rocky Hill, NJ, USA), IL-1β (1ng/ml; R&D Systems) and oncostatin M (OSM) (20ng/ml; Peprotech, Rocky Hill, NJ, USA), for 24 or 48 hours. This cytokine mix, called the IBD cytokine mix, was representative of the IBD microenvironment based on previous measurements in tissues of UC patients (41).

### RNA isolation and quantitative polymerase chain reaction

RNA from fibroblasts and tissues was extracted using the NucleoSpin RNA isolation kit (Macherey-Nagel, Düren, Germany) or Trizol reagent (Invitrogen), respectively, according to the manufacturer’s instructions. The concentration was measured with NanoDrop 1000 Spectrophotometer (Thermo Fisher Scientific, Waltham, US). Complementary DNA (cDNA) was synthesized by using RevertAid First strand cDNA synthesis kit (Thermo Fisher Scientific). qPCR was performed with SYBR Green Master mix (Bio-Rad laboratories, Nazareth, Belgium) using the iCycler Thermal Cycler and iQ5 Multicolour Real-Time PCR Detection System (Bio-Rad). Target genes were amplified using specific primers (**Supplementary Table 2**). Target gene expression levels were normalized to *ACTB* (*β-Actin*). The ΔCt method was applied to calculate the levels of gene expression, relative to the reference gene.

### Western blot analysis

Western blot analysis was performed as described before (42). In short, cells were lysed in radioimmunoprecipitation assay (RIPA) buffer after which the total protein content was determined using a detergent compatible (DC) protein assay (Bio-rad) according to the manufacturer’s instructions. Equal amounts of protein were separated with 10% polyacrylamide gel and transferred to PVDF membranes (Sigma). Non-specific binding was blocked with 5% milk in tris-buffered saline containing 0.5% Tween-20 (TBST, Merck, Darmstadt, Germany). Blots were incubated with rabbit anti-PFKFB3 (1:1000; Proteintech, Rosemont, USA), and mouse anti-β-Actin (1:1,000; Santa Cruz Biotechnology) for 2 hours at room temperature, followed by the incubation with appropriate horseradish peroxidase (HRP)-conjugated secondary antibodies (1:5,000; all from Agilent, CA, USA) for 1.5 hours at room temperature. Chemiluminescence (Thermo Fisher Scientific) was used to visualize the target proteins.

### Seahorse XF analyzer

Fibroblasts were seeded in 6-well plates at 100,000 cells per well and then stimulated with the cytokine mix described above, with or without the JAK/STAT inhibitor tofacitinib (5µM; Selleck Chemicals, Houston, USA). After 48 hours, the cells were detached, seeded in XFe96 cell culture plates (Seahorse Bioscience Europe, 102416-100) at a concentration of 20,000 cells per well, and incubated for 4 hours. Then the plate was incubated in a non-buffered, glucose-free RPMI-1640 medium (Sigma, R1383) for 1 hour in a CO_2_-free incubator. Cells were sequentially treated with the following inhibitors and activators: 10mM glucose, and 1μM oligomycin A (Cayman Chemical), which would effectively shut down oxidative phosphorylation (OXPHOS) and drive cells to use glycolysis to its maximum capacity, 3μM of fluoro-carbonyl cyanide phenylhydrazone (FCCP) (Sigma), a potent uncoupler of mitochondrial OXPHOS, and 1μM rotenone and antimycin A (Sigma), two compounds that ultimately lead to complete inhibition of mitochondrial OXPHOS. The extracellular acidification rate (ECAR) and oxygen consumption rate (OCR) were measured and used as a proxy for glycolytic rates and mitochondrial respiration. After the measurement, cells were washed with phosphate buffered saline (PBS) and lysed with RIPA-like buffer (50mM Tris hydrochloride (HCl), 150mM sodium chloride, and 0.5% sodium dodecyl sulfate (SDS), pH 7.4). Total protein concentration was measured for each well by DC protein assay. ECAR and OCR were normalized to the total protein levels in Wave (2.6.1).

### Colony formation and wound healing assays

Fibroblasts were seeded in 12-well plates (Coaster Corning Incorporated, 3512) at 1,000 cells per well. After 24 hours, the medium was replaced with fresh medium with or without the cytokine mix and/or PFKFB3 inhibitor PFK15 (1-2µM). The medium was refreshed every three days. After 2-3 weeks, cells were fixed with 2% PFA and stained with 0.05% crystal violet. Pictures were analyzed with ImageJ (U.S. National Institute of Health, USA). The percentage of the stained area was determined. Six independent experiments using different fibroblasts were performed.

For the wound healing assay, fibroblasts were seeded in 48-well plates (Corning Incorporated Costar, 3548) at 25,000 cells per well. When the fibroblasts were confluent, a scratch was made with a p200 pipet tip, and debris was removed by washing the cells once with the medium. Fresh medium (0% FCS) with or without cytokine mix and/or PFKFB3 inhibitor PFK15 (1-2µM) was then applied. Afterwards, pictures were taken with the Biotek Cytation5 (equipped with Biotek CO_2_ gas controller) at specific time points. The scratch area of each image was measured with ImageJ. Wound closure % = (A_0_ - A_t_)/A_0_ * 100% (A_0_ and A_t_ are the scratch area measured immediately and t hours after scratching, respectively). Four independent experiments using different fibroblasts were performed.

### Animal experiments

Animals were housed in ventilated cages and were given drinking water and food *ad libitum*. All the experiments were approved by the Dutch animal ethics committee and the Central Authority for Scientific Procedures on Animals (CCD).

For the dextran sulfate sodium (DSS)-induced colitis mouse model (43), 1.75% DSS (MV36,000-50,000 kDa, MP Biomedicals, IllKirch, France) was supplied to the drinking water of 8-week-old female C57BL/6 Jico mice (Charles River Laboratories, the Netherlands) for 8 days. The water was refreshed every two days. To evaluate the effects of PFKFB3 inhibition, mice were treated with PFK15, which was dissolved in dimethyl sulfoxide (DMSO) and injected intraperitoneally at a concentration of 25mg/kg every three days starting from the first day of DSS administration. Control mice received DMSO injections intraperitoneally. Mice were monitored daily for body weight, stool consistency, and colonic hemorrhage. The endoscopic colitis score (murine endoscopic index of colitis severity (MEICS)) was evaluated on day 8 under anesthesia with isoflurane. The MEICS score includes 5 parameters: thickening of the colon, changes of the vascular pattern, fibrin visible, granularity of the mucosal surface, and stool consistency. Each parameter can be scored 0~3, leading to a cumulative score between 0 (no signs of inflammation) and 15 (endoscopic signs of most severe inflammation) (44). After endoscopy, mice were sacrificed. Macroscopic inflammation score (including diarrhea, visible blood, and inflammation status), length and weight of the colon, were recorded after sacrificing the mice. Colon tissues were collected for further histology analysis.

The T-cell transfer model was established as previously described (45). In short, firstly, 8-week-old female C57BL/6 Jico donor mice were euthanized, and their spleens were harvested. Spleens were passed through a 70 µm cell strainer and erythrocytes were lysed with erythrocyte-lysis buffer (NH_4_Cl: 8.4g/L; KHCO_3_: 1g/L, pH = 7.4 ± 0.2, Pharmacy LUMC, Leiden, the Netherlands). Splenocytes were incubated with purified anti-mouse CD11b (clone: M1/70, Sony), purified anti-mouse CD45R/B220 (clone: RA3-6B2, Biolegend), and purified anti-mouse CD8a (clone: 53-6.7, Biolegend) for 30 minutes on ice. Next, they were incubated with Dynabeads (Invitrogen, Lithuania) for 30 minutes on a roller bank at 4°C. The mixture was then placed on the magnet, and the supernatant was collected and spun down at 400g for 7 minutes. Cells were then labeled with anti-CD4-PE-Cy5 and anti-CD45RB-FITC (BD Bioscience). 500,000 CD4+CD45RB^high^ T cells were sorted from the splenocytes using BD FACSAria flow cytometer and intraperitoneally injected into 9-week-old female B6.129S7-Rag1tm1Mom/J (Rag^-/-^) mice, which were bred in the animal breeding facility of the Leiden University Medical Center. Endoscopy was performed weekly to monitor the colitis starting from day 20. On day 27, mice were randomized into PFK15 treatment and vehicle control groups based on equal MEICS score. PFK15 was administered from day 28 *via* intraperitoneal injections every three days. The body weight, MEICS sore, macroscopic inflammation score, length and weight of colon were recorded on day 51 when the mice were sacrificed.

### Flow cytometry

Colons from the mice were minced with scissors and incubated in 375 µg/mL Liberase TL solution (Sigma-Aldrich, Zwijndrecht, the Netherlands) dissolved in Dulbecco’s Modified Eagle Medium (DMEM)/F12/Glutamax (Thermo Fisher Scientific, Paisley, UK) containing 10% fetal calf serum (FCS) (Thermo Fisher Scientific), for 30 minutes at 37°C. To obtain single cells, the suspension was filtered through Falcon™ Cell Strainers with 70 µm pore size (Corning, Durham, USA) and washed in fluorescence-activated single cell sorting (FACS) buffer (0.5% bovine serum albumin (BSA; Sigma), 0.02% NaN_3_ in PBS (Pharmacy LUMC, Leiden, the Netherlands)). Cells were stained with CD45.2-APC efluor 780 (1:400; Invitrogen, eBioscience), CD31-Biotin (1:800; Invitrogen, eBioscience), Epcam-PerCP-Cyanine 5.5 (1:800; Biolegend), and aqua live/dead (1:800; Thermo Fisher Scientific) for 45 minutes and then fixed with fixation concentrate (Thermo Fisher Scientific, Carlsbad, CA) for 1 hour at room temperature. Permeabilization buffer (Thermo Fisher Scientific, Carlsbad, CA) was used to permeabilize cells 2 times and rabbit anti-PFKFB3 (1:100; Proteintech, Rosemont, USA) and Brilliant Violet 605 Streptavidin (Biolegend) were applied for 45 minutes at room temperature, followed by Anti-Rabbit-Alexa 488 (Thermo Fisher Scientific) for 45 minutes. Cells were washed with FACS buffer and measured on the Fortessa (BD bioscience, Vianen, the Netherlands). Flow cytometry data analysis was performed using Flowjo 10 software (BD bioscience, Vianen, the Netherlands). Dead cells, hematopoietic cells (CD45), endothelial cells (CD31), and epithelial cells (Epcam) were excluded to get the stromal cell population (**Figure S2**).

### Tissue processing and histological analysis

Tissues were fixed and processed in the Leica TP1020 tissue processor (Leica, Amsterdam, The Netherlands) and subsequently embedded in paraffin. For hematoxylin and eosin staining, slides were deparaffinized and rehydrated and then stained with hematoxylin for 5 minutes. After washing with tap water, the slides were stained with eosin for 1 minute and dehydrated before being mounted with Entellan (Merck KGaA, Darmstadt, Germany). Images were made with the slide scanner (3DHISTECH panoramic 250). The IBD histology score was calculated based on the immune cell infiltrates, crypt architecture, muscle thickening, and goblet cell depletion (46). The score was performed by two individuals who were blinded to treatment groups.

For immunohistochemistry (IHC) and immunofluorescence (IF), paraffin sections (4µm) were deparaffinized and rehydrated, after antigen retrieval was performed by 10 min boiling in 0.01M sodium citrate buffer, pH6.0. Slides were then incubated with primary antibodies, rabbit anti-PFKFB3 (1:1600; Proteintech, Rosemont, USA), rat anti-CD45 (1:3200; eBioscience, San Diego, USA), goat anti-CD105 (1:800; R&D, Minneapolis, USA), mouse anti-α-SMA (1:3200; Progen, Heidelberg, Germany), rabbit anti-vimentin (1:400; Cell signaling, Massachusetts, USA), and mouse anti-human THY1 (1:5000; Novus-Bio techne, Minneapolis, Minnesota, USA) diluted in 1% bovine serum albumin (BSA) in PBS overnight at 4°C. Afterward, the appropriate secondary biotinylated antibodies (Agilent technologies, CA, USA) or anti-mouse-Alexa 488 (Thermo Fisher Scientific) and anti-Rabbit-Alexa 568 (Thermo Fisher Scientific) were applied (1:200, 30 min at room temperature). For IHC staining, this was followed by incubation with Vectastain (Vector Laboratories, CA, USA) for 30 minutes and visualization with Dako Liquid DAB + Substrate Chromogen System (Agilent Technologies) for 10 minutes at room temperature. The slides were counterstained with hematoxylin, and then dehydrated and mounted. Images were made with the slide scanner (3DHISTECH panoramic 250). To quantify the IHC staining, the whole colon was annotated with QuPath-0.3.2. Positive staining was then recognized, and the percentage of the positive area was calculated. For IF staining, Slowfade Gold Antifade reagent with DAPI (Invitrogen, Waltham, USA) was applied. Images were made with the slide scanner (Zeiss Axio ScanZ1).

To visualize total collagen content, slides were stained with 0.1% Sirius red in saturated picric acid for 90 minutes, after which, they were washed in 0.01N hydrochloric acid for 5 minutes. Slides were further washed with Millipore water, dehydrated, and mounted. Images were made with the slide scanner (3DHISTECH panoramic 250). To quantify the collagen staining, the whole colon was annotated with QuPath-0.3.2. Positive staining was then quantified as the percentage of the stained area.

### Statistical analysis

Data were presented as means ± standard deviation from representative experiments or independent replicates which are indicated in the figure legend. The non-paired/paired Student t tests, Mann-Whitney test, or Wilcoxon tests were used to compare 2 groups. Differences between more than 2 groups were measured using one-way analysis of variance (ANOVA) or Kruskal–Wallis test followed by Dunn’s multiple comparisons test. All analyses were performed using GraphPad Prism software (San Diego, CA, USA). P values of 0.05 or less were considered statistically significant.

## Results

### Increased glycolysis and PFKFB3 expression in IBD samples

**Figure 1:**
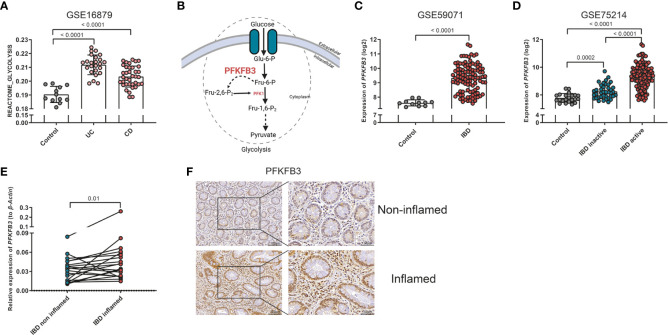
Elevated glycolysis and PFKFB3 expression in IBD samples. **(A)** ssGSEA analysis of GSE16879, glycolysis score of each sample is displayed in the graph (Control, n=12 (normal intestine), UC, n=24, CD, n=37). **(B)** Schematic overview on the role of PFKFB3 in the glycolysis process. **(C)** *PFKFB3* expression in dataset GSE59071 (Control, n=11, IBD, n=105). **(D)** *PFKFB3* expression in dataset GSE75214 (Control, n=22, IBD inactive, n=39, IBD active, n=133). **(E)** Relative expression of *PFKFB3*, corrected for *β-Actin* as a housekeeping gene in LUMC-IBD biobank tissues (n=20, paired samples, non-inflamed/inflamed). **(F)** Representative immunohistochemical staining for PFKFB3 in human IBD tissues (n=9, paired sample, non-inflamed/inflamed, scale bar, 100μm (left) or 50μm (right)). The data are presented as the mean ± SD. One-way ANOVA **(A, D)**, non-paired two-tailed t-test **(C)**, and Wilcoxon test **(E)** were performed.

Firstly, we investigated the role of glycolysis in IBD, using online expression databases. Single sample gene set enrichment analysis (ssGSEA) in 7 out of 8 databases indicated that glycolysis was increased in IBD samples, both in UC and CD, when inflamed tissues were compared to healthy control or non-inflamed intestinal tissues (**Figures 1A**, **S3A-G**). Given the central role of PFKFB3 in enhancing glycolytic rates (**Figure 1B**), we then explored the expression of PFKFB3 in IBD samples. It was revealed that *PFKFB3* was expressed at significantly higher levels in (inflamed) IBD tissues when compared to controls or non-inflamed tissues (**Figures 1C, D**). These results were confirmed in samples from the LUMC-IBD biobank showing elevated expression of *PFKFB3* in inflamed compared to paired non-inflamed tissues from IBD patients (**Figure 1E**). Next, we evaluated the cellular localization of PFKFB3 in inflamed IBD tissues. Immunohistochemistry showed PFKFB3 expression in several cell types, including stromal cells. Importantly, we observed increased PFKFB3 expression in inflamed tissue from IBD patients compared to matched non-inflamed tissues (**Figure 1F**, negative control in **Figure S3H**). Taken together these data show increased PFKFB3 expression in inflamed tissues from IBD patients.

### PFKFB3 expression is elevated in IBD stromal cells

**Figure 2:**
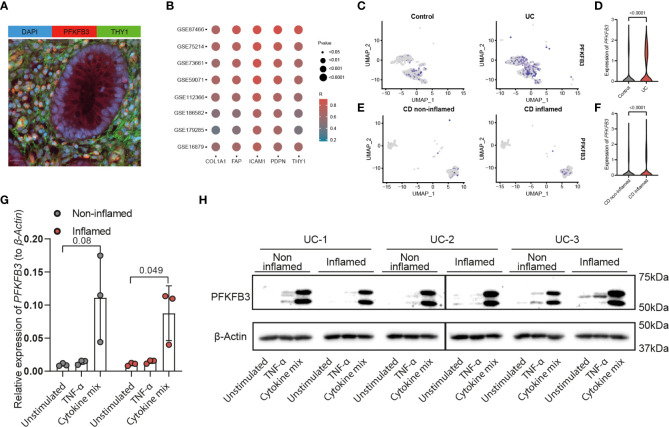
PFKFB3 expression was associated with stromal marker gene expression and elevated in fibroblasts in an inflammatory environment. **(A)** Immunofluorescent staining of PFKFB3 and the fibroblast marker THY1 in IBD tissue (PFKFB in red; THY1 in green; DAPI in blue (nuclei). Scale bar is 20μm. **(B)** Spearman’s correlation of *PFKFB3* expression and stromal genes (*COL1A1*, *FAP*, *ICAM1*, *PDPN*, and *THY1*) expression in 8 online datasets. Colors represent the level of correlation, dot size indicates the p value. UMAP plot of stromal cells in datasets GSE114374 **(C)** and GSE134809 **(E)**. PFKFB3 positive stromal cells are indicated in purple in healthy controls **(C,** left**)**, UC **(C,** right**)** colon and in non-inflamed **(E,** left**)** and inflamed **(E,** right**)** ileum from CD patients. In GSE114374, exclusion markers including *PECAM1*, *S100B*, *RGS5*, and *SDC1*, were used to exclude the other cell types. In GSE134809, markers for fibroblasts (*CCL13*, *CCL8*) and activated fibroblasts (*CXCL8*, *CXCL3*, *CXCL1*, and *CXCL6*) were used to identify stromal cells. **(D, F)** Violin plots of expression of PFKFB3 in stromal cells of GSE114374 **(D)** and GSE134809 **(F)**. **(G, H)** Primary fibroblasts (n=3, including inflamed and non-inflamed sites for each patient) were stimulated with TNF-α (10ng/ml) and cytokine mix (IL17-A: 50ng/ml, OSM: 20ng/ml, IL-1β: 1ng/ml) for 24 **(G)** or 48 hours **(H)**. PFKFB3 expression was determined with qPCR analysis **(G)** and western blot **(H)**. β-Actin was used as a housekeeping gene. Mann-Whitney and one-way ANOVA statistical tests were performed.

In order to evaluate if PFKFB3 is expressed in stromal cells, an immunofluorescent co-staining of PFKFB3 and THY1, a general stromal cell marker, was performed. The result shows that PFKFB3 expression colocalized with THY1 expression in IBD tissues (**Figure 2A**). To further substantiate these findings, Spearman’s correlation analysis was performed to investigate the relation between the expression of *PFKFB3* and stromal genes, including collagen type 1 alpha 1 chain (*COL1A1*), fibroblast activation protein-α (*FAP*), podoplanin (*PDPN*), *THY1* and intercellular adhesion molecule 1 (*ICAM1*). These data show that *PFKFB3* expression was positively correlated with all these stromal genes in the 8 independent datasets indicated above (**Figure 2B**). Next, we evaluated if this was due to overall increased expression or an increased number of PFKFB3 expressing cells. By analyzing the scRNA sequencing datasets, GSE114374 and GSE134809, we found that there were indeed significantly more *PFKFB3*-expressing stromal cells (**Figures 2C, E**, **S4**) and, additionally, higher stromal *PFKFB3* expression was detected (**Figures 2D, F**) in UC patients and inflamed CD patients compared to healthy control and non-inflamed CD samples, respectively. Expression of *PFKFB3* was also detected in endothelial cells, glial cells, pericytes, and plasma cells, with the stromal cells showing the highest expression (**Figure S5**). These data showed that stromal cells are one of the main cell types which express a high level of PFKFB3.

To explore the levels and regulation of PFKFB3 expression *in vitro*, fibroblasts were isolated from endoscopic biopsies from UC patients, from inflamed and non-inflamed sites. In contrast to fresh fibroblasts, cultured fibroblasts showed expression of PFKFB3 independent of their origin (inflamed/non-inflamed, **Figure 2G**). Next, we investigated if local inflammation could regulate PFKFB3 expression. To mimic the local inflammatory environment *in vitro*, we stimulated the primary fibroblasts with TNF-α, or a cytokine mix mimicking the local UC environment, consisting of IL-17A, OSM, and IL-1β. The composition of this cytokine mix was based on the levels of these cytokines detected in UC patient tissues (41). qPCR showed that *PFKFB3* mRNA expression was strongly increased (5-20x) after the stimulation with the UC cytokine mix (**Figure 2G**). Western blot analysis confirmed the increased protein expression of PFKFB3 after the stimulation (**Figure 2H**). Taken together, these data indicate that fibroblasts strongly increase PFKFB3 expression under (UC-like) inflammatory conditions.

### Fibroblasts glycolytic activity is increased in an inflammatory environment

**Figure 3:**
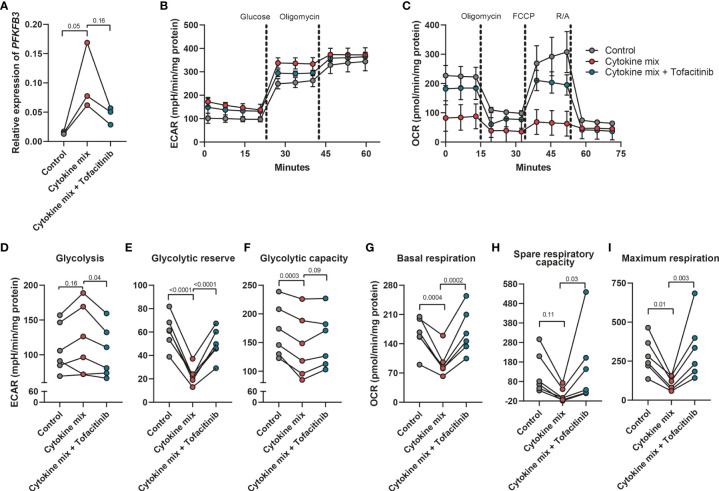
Increased glycolytic level in fibroblasts after the stimulation with inflammatory cytokines. Primary fibroblasts (n=6 independent fibroblasts) were stimulated with cytokine mix (IL17-A: 50ng/ml; OSM: 20ng/ml; IL-1β: 1ng/ml) with/without tofacitinib (5μM) for 48 hours and collected to perform the seahorse XFe96 experiment. The remaining cells were used for qPCR analysis. **(A)** qPCR analysis of the expression of PFKFB3 after the stimulation with cytokine mix with/without tofacitinib for 48 hours. **(B, C)** Representative seahorse curves for ECAR and OCR of primary fibroblasts. ECAR values were analyzed in response to the injection of glucose (10mM) and oligomycin (1μM) whereas OCR values were analyzed in response to the injection of oligomycin (1μM), FCCP (3μM), and rotenone and antimycin A (R/A; 1μM). Data were normalized to the total protein level (DC protein assay) and are presented with mean ± SD from a representative experiment. **(D–I)** Glycolysis **(D)**, glycolytic reserve **(E)**, glycolytic capacity **(F)**, baseline mitochondrial respiration **(G)**, spare respiratory capacity **(H)**, and maximum respiration **(I)** were calculated and presented, n=6 fibroblasts. One-way ANOVA comparisons test was performed.

Given the observed upregulation of PFKFB3 by fibroblasts in an inflammatory environment, we hypothesized that this increase could lead to an increased glycolytic activity in these cells. To evaluate this hypothesis, cells were seeded and treated with the UC cytokine mix, with or without tofacitinib, an inhibitor of Janus Kinases (JAKs), a central mediator of the inflammatory signaling pathway (47). Fibroblasts exposed to the UC cytokine mix showed upregulation of *PFKFB3*, while inhibition of JAK signaling pathway reverted this modulation (**Figure 3A**). Concomitant with the increase in *PFKFB3* expression, we observed an increase in glycolysis after stimulation with the UC cytokine mix compared to control, which was also reverted when the inhibitor tofacitinib was added (**Figures 3B, D**). Oppositely, glycolytic reserve and glycolytic capacity were decreased under inflammatory conditions and increased again upon inhibition of inflammation (**Figures 3E, F**). This might suggest that under inflammatory conditions, fibroblasts are already close to their maximum glycolytic metabolism. In agreement with the increased glycolytic activity observed under inflammatory conditions, baseline mitochondrial respiration, spare respiratory capacity (SRC), and maximum respiration were decreased when fibroblasts were exposed to the UC cytokine mix which was reversed by the inhibition of inflammation (**Figures 3C, G-I**). Taken together, these data suggest that fibroblasts exposed to inflammatory conditions shift their metabolism from OXPHOS toward glycolysis in a JAK-dependent manner and that this metabolic reprogramming is associated with the expression of PFKFB3.

### PFKFB3 inhibition impairs the migration and proliferation of fibroblasts

**Figure 4:**
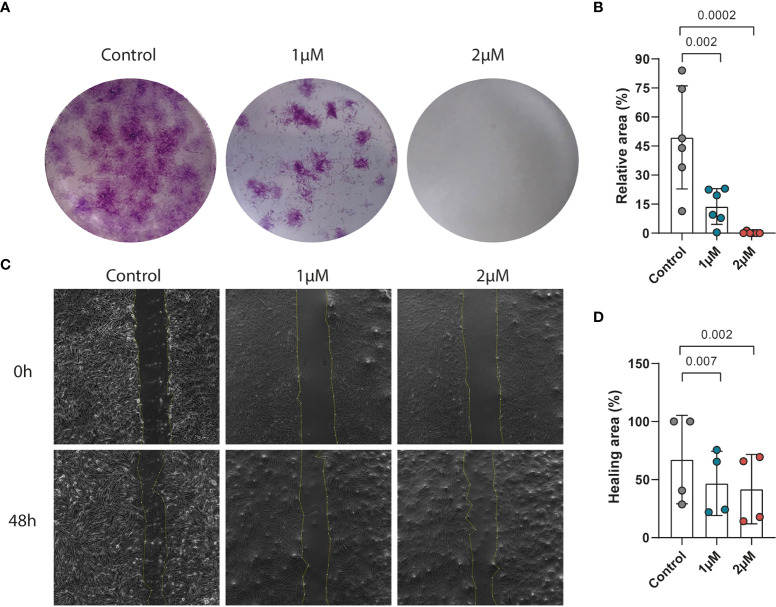
PFKFB3 inhibition impaired the migration and proliferation of fibroblasts. Primary fibroblasts were treated with PFK15 (1μM or 2 μM) and the effects on proliferation and migration were studied. **(A)** Representative image from the colony formation assay, showing strongly decreased proliferation upon PFKFB3 inhibition. **(B)** Bar graph represented the statistical results of the relative colony area (n=6 independent fibroblast). **(C)** Representative phase-contrast microscope images showing the area covered by the cells at 0 and 48 h after wounding, showing decreased wound healing upon PFK15 treatment. **(D)** Quantification of the wound-healing assay. The data were normalized to the wound width of each group at 0 h (n=4 independent fibroblasts). One-way ANOVA was performed.

Previously it has been reported that activated fibroblasts have an increased proliferation and migration rate in CD (48). To explore the effect of PFKFB3 inhibition on the proliferation of fibroblasts, a colony formation assay was performed with increasing concentrations of PFK15, a selective inhibitor of PFKFB3. These results showed that inhibition of PFKFB3 led to a concentration-dependent decrease in fibroblast proliferation (**Figures 4A, B**). Furthermore, PFKFB3 inhibition attenuated the migration of fibroblasts in a wound healing assay (**Figures 4C, D**). Stimulation of the fibroblasts with the IBD cytokine mix did not significantly affect their proliferation while inhibition of PFKFB3 could still decrease fibroblast proliferation in the inflammatory environment (**Figures S6A, B**). Inflammatory fibroblasts surprisingly led to the attenuation of the migration of the fibroblasts which was not affected by inhibition of PFKFB3 (**Figures S6C, D**). These data show that PFKFB3-dependent glycolysis is involved in wound repair upon injury.

### PFKFB3 inhibition decreases the expression of pro-inflammatory cytokines and chemokines in fibroblasts

**Figure 5:**
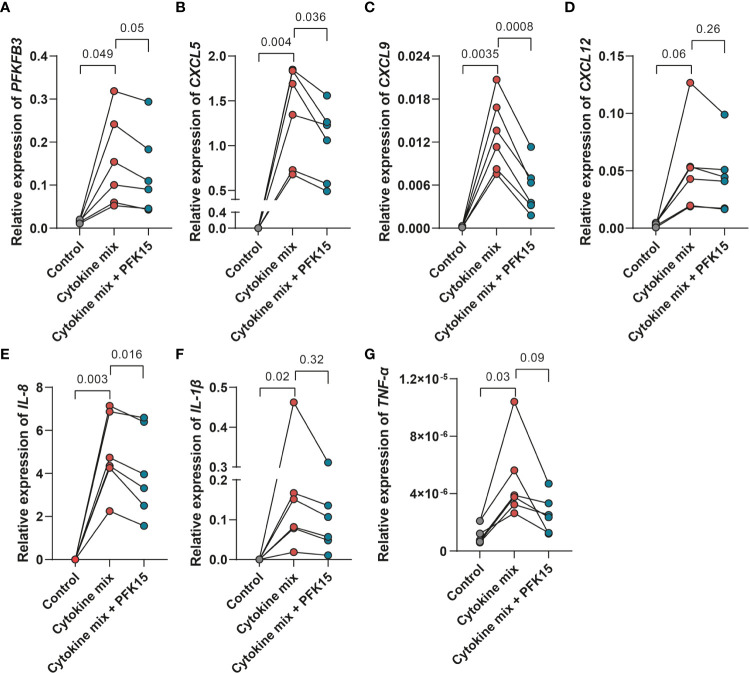
PFKFB3 inhibition decreased the expression of pro-inflammatory cytokines and chemokines in fibroblasts. Primary fibroblasts (n=6 independent fibroblasts) were stimulated with the UC cytokine mix (IL17-A: 50ng/ml; OSM: 20ng/ml; IL-1β: 1ng/ml) with or without PFK15 (1μM) for 24 hours and collected for qPCR analysis. Relative expression (to *β-Actin*) of *PFKFB3* **(A)**, *CXCL5* **(B)**, *CXCL9* **(C)**, *CXCL12* **(D)**, *IL-8* **(E)**, *IL-1β* **(F)**, and *TNF-α* **(G)** was determined. One-way ANOVA was performed to test for significance.

Inflammatory cytokine production is supported by glycolysis in many cells. This made us wonder whether PFKFB3, through control of glycolysis, may also regulate cytokine expression by stromal cells. Firstly, we investigated the relationship between *PFKFB3* expression and immune cell infiltration in IBD samples. Spearman’s correlation analyses were performed to assess the relation between *PFKFB3* expression and the number of immune cells in intestinal tissues. To select highly correlated immune cells, we set absolute Spearman’s correlation > 0.4. The results showed that PFKFB3 expression was significantly positively correlated with the infiltration of activated dendritic cells (aDCs), macrophages, neutrophils, and Th1 cell infiltration in all datasets (**Figure S7**). Since fibroblasts highly express *PFKFB3* under inflammatory conditions, we explored if *PFKFB3* could influence inflammatory cytokine expression in intestinal fibroblasts. We stimulated fibroblasts with the UC cytokine mix with/without PFK15 and analyzed the expression of pro-inflammatory cytokines and chemokines. Interestingly, we found that the expression of chemokines like *CXCL5*, *CXCL9*, and *CXCL12*, and pro-inflammatory cytokines like *TNF-α*, *IL-1β*, and *IL-8*, was increased after stimulation with the UC cytokine mix and consistently reduced by treatment with PFK15, which has the same expression pattern with *PFKFB3* (**Figure 5**). These data indicate that fibroblasts could cause increased immune cell infiltration in IBD by upregulation of chemoattractants and that stromal PFKFB3 might be an important regulator.

### PFKFB3 inhibition attenuates colitis severity in experimental colitis

To explore if inhibition of PFKFB3 could alleviate colitis *in vivo*, first, the expression of Pfkfb3 in mice colon was evaluated. qPCR analysis showed that *Pfkfb3* is expressed higher during colitis compared to healthy controls in the T cell transfer model for colitis and dextran sulfate sodium (DSS)-induced colitis (**Figure S8A**). Flow cytometry data also showed that the percentage of PFKFB3 positive cells and the mean fluorescence intensity increased in stromal cells in DSS-induced colitis (**Figures S8B, C**).

**Figure 6:**
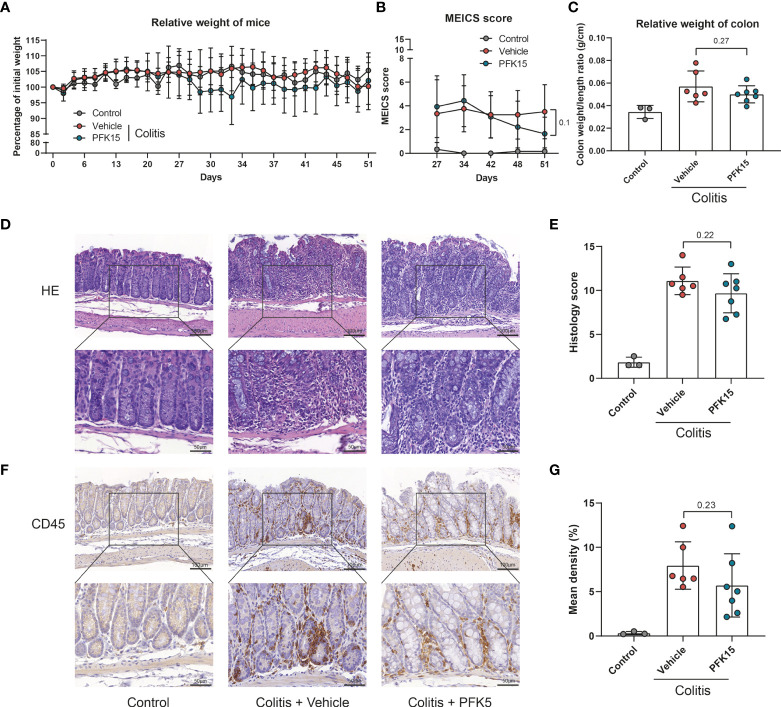
Inhibition of PFKFB3 attenuated inflammation in T cell transfer colitis. Rag^-/-^ were received 500,000 CD4+CD45RB^high^ T cells or PBS (control group, n=3). Upon development of colitis at around day 27, they were randomized as PFK15 (n=7), or vehicle (n=6) group based on equal average MEICS score. PFK15 (25mg/kg) or DMSO (equal volume) was injected intraperitoneally every three days. **(A)** Body weight of mice expressed as the percentage of body weight at day 0. **(B)** MEICS score of the mice in every group at day 27, -34, -42, -48 and -51 (sacrifice). **(C)** Colon weight/length ratio (g/cm) of the mice upon sacrifice. **(D)** Representative hematoxylin and eosin staining of the colon. **(E)** Quantification of IBD histology score of the colon. **(F)** Representative immunohistochemical staining for the immune cell marker CD45. **(G)** Quantification of the percentage of CD45 positive stained area. Unpaired two-tailed t-test was performed to assess statistical significance between vehicle and treated mice. Scale bar, 100μm (upper panel) or 50μm (lower panel).

For T cell transfer model, colitis was induced with intraperitoneal injection of CD4+CD45RB^high^ T cells from donor mice. After 27 days, mice were randomized into PFK15 treatment and vehicle control groups based on equal MEICS score. No significant change in body weight of the mice was observed (**Figure 6A**), while the MEICS score tended to decrease in the PFK15-treated mice on day 51 (1.64 vs 3.5, p = 0.1, **Figure 6B**). The colon weight/length ratio was slightly decreased, although not reaching statistical significance (0.050 vs 0.057 g/cm, p = 0.27, **Figure 6C**). Histological analysis showed a lower histology score, although not reaching statistical significance, in the PFK15 treated group compared with vehicle control (9.68 vs 11.08, p = 0.22, **Figure 6D, E**). Immunohistochemical staining of CD45 also revealed fewer immune cells in 4 out of 6 colons of mice treated with PFK15 (5.701% vs 7.943%, p = 0.23, **Figures 6F, G**). These data indicate that PFKFB3 inhibition by PFK15 can reduce T-cell transfer colitis, but not all mice respond to the treatment.

**Figure 7:**
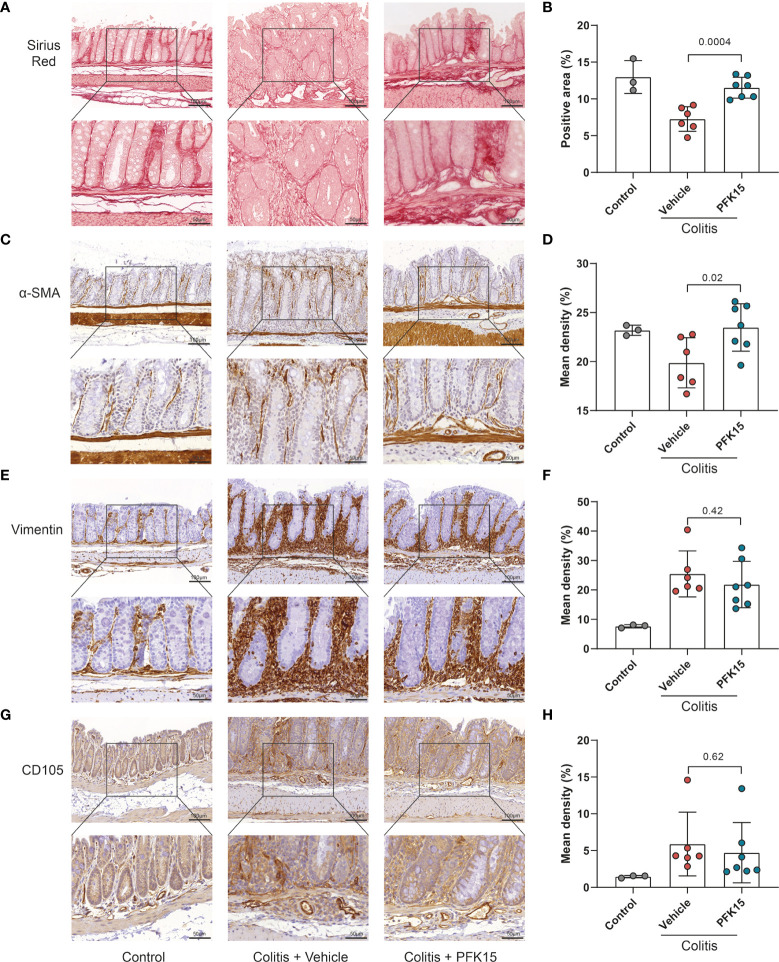
Inhibition of PFKFB3 restores the stromal in T cell transfer colitis. **(A)** Representative image from Sirius red staining of the colon. **(B)** Quantification analysis, based on the percentage of the positively stained area. Representative immunohistochemical staining of α-SMA **(C)**, vimentin **(E)**, and CD105 **(G)** with quantification analysis, based on the percentage of the positively stained area in the total area **(D, F, H).** Unpaired two-tailed t-test was performed to assess statistical significance between vehicle treated and PFK15 treated mice. Scale bar, 100μm (upper panel) or 50μm (lower panel).

To evaluate changes in the stromal compartment upon PFKFB3 inhibition by PFK15, total collagen content was evaluated using Sirius red staining, combined with immunohistochemistry for α-SMA, as a marker for a subset of activated fibroblasts, the mesenchymal marker vimentin, and CD105 (endoglin), a marker for angiogenesis. Compared to the vehicle group, increased collagen and α-SMA were detected in the intestines of mice from the PFK15 group (**Figures 7A-D**). Four out of six mice showed decreased vimentin staining in the PFK15 treated group (**Figures 7E, F**), while CD105 expression did not change (**Figures 7G, H**).

**Figure 8:**
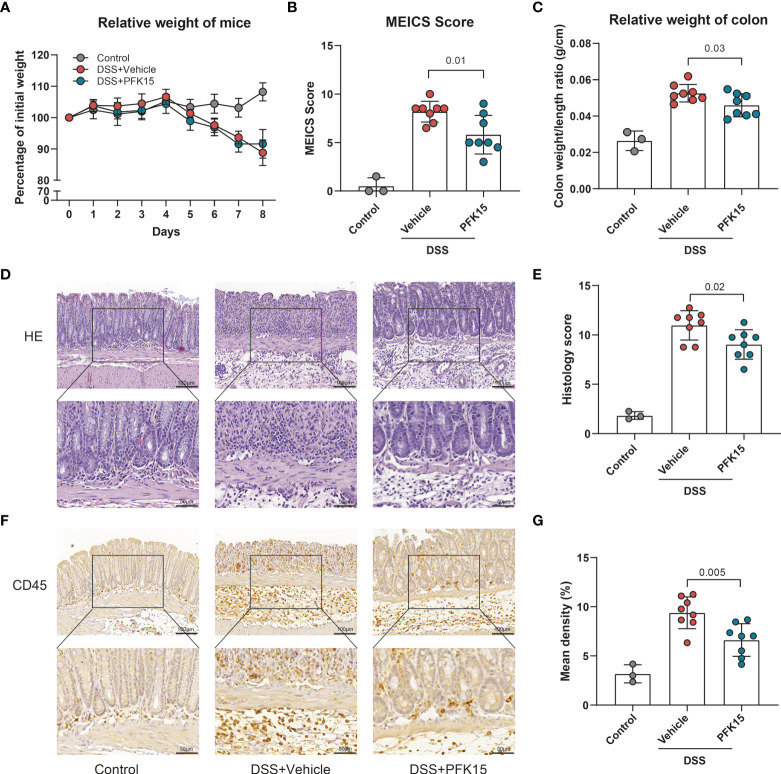
Inhibition of PFKFB3 attenuated inflammation in DSS-induced colitis. Mice received 1.75% DSS (n=3 control, n=16 DSS) at the start of the experiment. From day 1, DSS mice were treated with PFK15 (25mg/kg) or DMSO (n=8/group) every three days. **(A)** Body weight of mice was measured daily and expressed as the percentage of body weight on day 0. **(B)** MEICS score of the mice on day 8. **(C)** Colon weight/length ratio (g/cm) of the mice. **(D)** Representative hematoxylin and eosin staining of the colon. **(E)** Quantification of IBD histology score of the colon. **(F)** Representative immunohistochemical stainings of immune cell marker CD45. **(G)** Quantification of the percentage of CD45 positive stained area. Unpaired two-tailed t-test was performed to assess statistical significance between vehicle treated and PFK15 treated mice. Scale bar, 100μm (upper panel) or 50μm (lower panel).

To further substantiate these findings, we employed another mouse model for IBD, DSS-induced colitis. Colitis was induced with DSS applied *via* the drinking water. Mice were simultaneously treated with PFK15 or vehicle (DMSO) *via* intraperitoneal injections. DSS strongly decreased the body weight of the mice (**Figure 8A**) and treatment with PFK15 did not significantly affect body weight loss but did decrease the endoscopic colitis (MEICS) score significantly (5.8 vs 8.2 in vehicle-treated mice, p = 0.01, **Figure 8B**). This was accompanied by a decrease in colon weight/length ratio (0.046 vs 0.053 g/cm, p = 0.03, **Figure 8C**). Histological evaluation of the colon indicated decreased histology score upon treatment with PFK15 compared to the vehicle group in DSS-induced colitis (9.03 vs 10.97, p = 0.02, **Figures 8D, E**). Immunohistochemical analysis also showed a decreased number of infiltrating CD45+ cells in PFK15 treated mice (6.622% vs 9.392%, p = 0.0045, **Figures 8F, G**). Taken together these data show that PFKFB3 inhibition can reduce DSS-induced colitis in mice.

**Figure 9:**
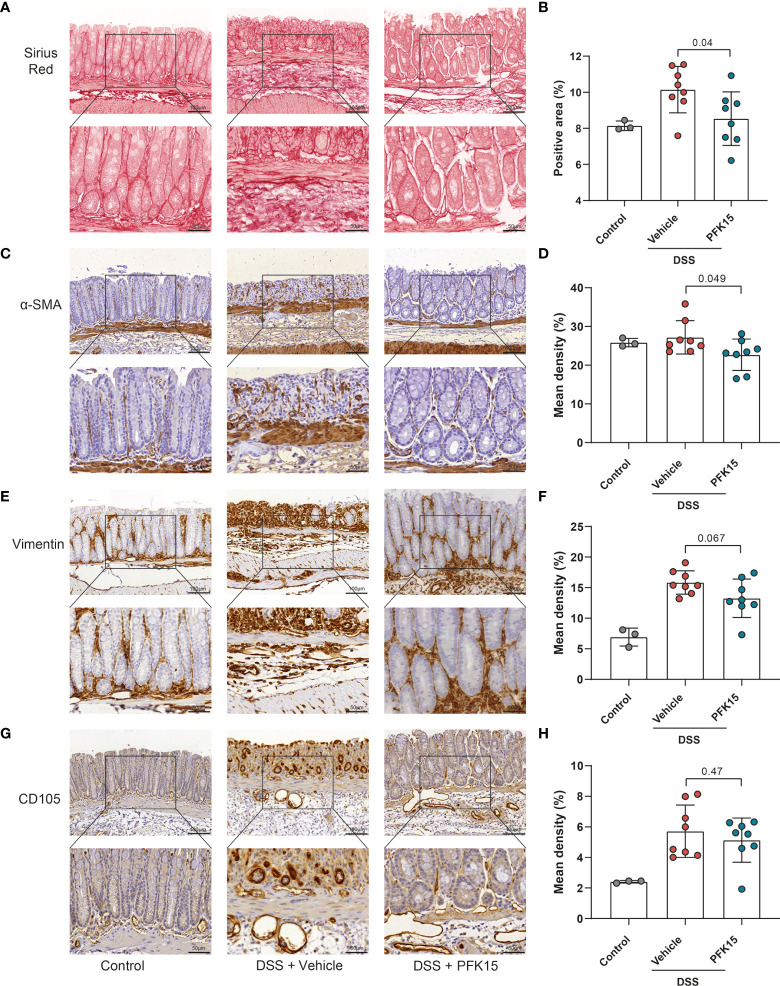
Inhibition of PFKFB3 decreased the stromal activation in DSS-induced colitis. **(A)** Representative image from Sirius red staining of the colon. **(B)** Quantification analysis, based on the percentage of the positively stained area. Representative images for staining of α-SMA **(C)**, vimentin **(E)**, and CD105 **(G)** with quantification analysis, based on the percentage of positively stained area in the total area **(D, F, H)**. Unpaired two-tailed t-test was performed to assess statistical significance between vehicle treated and PFK15 treated mice. Scale bar, 100μm (upper panel) or 50μm (lower panel).

For the stromal compartment upon PFKFB3 inhibition by PFK15, as shown in **Figure 9**, less collagen (**Figures 9A, B**), fewer α-SMA expressing cells (**Figures 9C, D**), and fewer vimentin positive cells (**Figures 9E, F**) were observed following PFK15 treatment in DSS-induced colitis. CD105 expression did not differ significantly between PFK15 and vehicle treated groups (**Figures 9G, H**). Taken together these data reveal that inhibition of PFKFB3 activity reduces stromal accumulation, activation, and collagen deposition, accompanied by decreased intestinal inflammation in a DSS- induced colitis model.

## Discussion

Our study showed, for the first time, that PFKFB3 expression is highly increased in human IBD intestinal samples, being expressed by stromal fibroblasts and upregulated under inflammatory conditions in this cell type. This upregulation leads to higher glycolysis and increased expression of chemoattractants. Concordantly, inhibition of PFKFB3 decreases the expression of chemokines and pro-inflammatory cytokines in intestinal fibroblasts and decreased colitis severity in two mouse models for IBD. Our findings suggest that PFKFB3 is important to the pro-inflammatory phenotype of fibroblasts, and thereby might contribute to the pathogenesis of IBD.

Glycolysis is the process in which glucose converts into pyruvate accompanied by the synthesis of adenosine triphosphate (ATP) and metabolic intermediates (49). Our study shows that glycolytic levels are increased in IBD samples, accompanied by an increased stromal expression of PFKFB3, one of the key enzymes involved in glycolysis. Consistent with our finding, Vermeulen and colleagues reported that multiple glycolytic enzymes, including aldolase A, phosphoglycerate mutase, alpha-enolase, triosephosphate isomerase, and malate dehydrogenase, showed strong seroreactivity in IBD patients using the immunoproteomic approach. The detection rate of at least one of these five antigens was 53.3% in UC, 38.3% in CD, and 8.3% in healthy controls, indicating increased glycolytic activity in IBD patients. Furthermore, the mRNA expression of hypoxia-inducible factor 1 alpha (HIF-1α), which can regulate these glycolytic enzymes, was also increased in colonic samples from UC and CD patients compared to healthy controls (50). Additionally, pyruvate kinase M2 (PKM2), an enzyme that catalyzes the last step within the glycolysis pathway, was elevated in serum and fecal samples from patients with IBD (51). In murine colitis, including DSS-induced and IL-10- deficiency colitis, metabolomics revealed a significant increase in lactate levels and reduction in the proteins involved in oxidative phosphorylation, which implies that the respiratory state of the colon would switch to anaerobic metabolism because of the inflammation-induced hypoxia (52). All these studies support the notion that glycolysis is increased in colitis. Furthermore, as a key regulator of glycolysis, PFKFB3 has been regarded as a promising target for the treatment of multiple tumors, rheumatoid arthritis, pulmonary arterial hypertension, and atherosclerosis (23, 28, 53, 54). Our data now show that inhibition of PFKFB3 can also reduce colitis in mice and restore intestinal stromal homeostasis.

Stromal glycolysis has been implicated in the pathogenesis of several other inflammatory diseases. In rheumatoid arthritis, there is growing evidence that synovial fibroblasts rely on high glycolysis and inhibition of PFKFB3 alleviates synovial fibroblast-mediated synovial inflammation (22, 23, 55). Single-cell RNA sequencing analysis showed that in IBD the stromal expression of PFKFB3 in intestinal fibroblasts is strongly increased in inflamed tissues. However, cultured fibroblasts showed equal expression of PFKFB3 independent of their origin (inflamed/non-inflamed), which indicates that cultured fibroblasts are distinct from *in vivo* fibroblasts. Similarly, Stalmann and colleagues also reported the difference between cultured bone marrow stromal cells and *in vivo* mesenchymal stromal cells (56). Importantly exposing the fibroblasts to a “UC-like” inflammatory environment (41) strongly increases PFKFB3 expression, reflecting the expression pattern observed in tissues. Furthermore, during such an inflammatory state, fibroblasts have a higher glycolytic rate. This could be reverted by the inhibition of the main inflammatory pathway using a JAK/STAT-inhibitor. Interestingly fibroblasts express higher pro-inflammatory cytokines, like *IL-8*, *TNF-α*, *IL-1β*, and some chemokines, such as *CXCL5*, *CXCL9*, and *CXCL12*, under inflammatory conditions. Importantly this was dependent on PFKFB3 activity as inhibition of PFKFB3 decreased the secretion of pro-inflammatory cytokines by fibroblasts. All these results indicate that PFKFB3 plays a central role in the inflammatory metabolic reprogramming of fibroblasts. Previously, a link between PFKFB3 expression and inflammatory reaction/immune cell recruitment has been made. In endothelial cells, knockdown of PFKFB3 inhibited TNF-α-induced monocyte adhesion and transmigration to endothelial cells, and PFKFB3 inhibition interfered with nuclear factor kappa B (NF-κB) pathway activation (57). In macrophages, the activation of nucleotide-binding domain-like receptor Family Pyrin Domain Containing 3 (NLRP3) inflammasome and subsequent release of IL-1β are regulated by glycolysis *via* PFKFB3 (58). This might indicate that PFKFB3 is important for the metabolic identity of immune cells. Simultaneously, increased expression of PFKFB3 in fibroblast-like synoviocytes might activate NF-κB and the mitogen-activated protein kinases (MAPKs) pathways, which has been linked to synovial inflammation (23). All these findings show that PFKFB3 plays a vital role in the immunoregulation in various cell types and our current work extends these observations to intestinal fibroblasts.

To better understand how PFKFB3 could modulate intestinal inflammation, we explored its role by taking advantage of experimental models of colitis, such as DSS and T cell transfer. DSS-induced colitis starts with the damage of the colonic epithelial layer, followed by stimulating local inflammation, which manifests in increased pro-inflammatory cytokines from epithelial and immune cells, like TNF-α, IL-1β, and IL-10 (59, 60). Injury and inflammation result in the proliferation of stromal cells which facilitate wound repair in the acute phase (61). Our results show that inhibition of PFKFB3 indeed hampers the inflammation in DSS-induced colitis, simultaneously also decreasing the number of activated/fibrotic stromal cells (highlighted by reduced levels of collagen, α-SMA, and vimentin). These data imply that PFKFB3 plays an essential role in inflammation, potentially partly *via* modulating the stromal cell phenotype. Furthermore, it is tempting to speculate that, based on our *in vitro* observations, the secretion of pro-inflammatory cytokines by stromal cells is decreased. Glycolytic reprogramming was also reported in several fibrotic diseases, such as lung fibrosis, cardiac fibrosis, and renal fibrosis, and targeting glycolysis alleviated fibrotic disorders both *in vitro* and *in vivo* (62–65). As a common and serious complication of IBD, intestinal fibrosis is an inevitable consequence of chronic tissue injury (66). Given the data we report, inhibition of PFKFB3 could attenuate this fibrotic response, although it must be noted that glycolysis and stromal remodeling is required for tissue repair.

Current IBD therapies such as corticosteroids, and 5-aminosalicylate, modulate colitis by blocking mammalian target of the rapamycin complex 1 (mTORC1), which results in decreasing HIF-1α expression and thereby glycolysis (67, 68). This indicates that part of their clinical effects might be through regulating glycolysis. Other therapeutic molecules, targeting glycolytic enzymes, also facilitate eliciting an anti-inflammatory effect (69). For example, 2-deoxy-d-glucose (2DG) has been shown to be effective in the inhibition of inflammation in several mouse models, such as TC lupus model, similar to systemic lupus erythematosus (SLE) (70), K/BxN (KBN) mouse model of arthritis (71), and experimental autoimmune neuritis (EAN) rats for Guillain-Barré syndrome (GBS) (72). Another inhibitor, CG-5, inhibiting glucose transporters, is reported to ameliorate autoimmune phenotypes in TC lupus model, partially *via* the inhibition of glycolysis in CD4+ T cells (73).

Inhibition of PFKFB3 by PFK15 has been reported to alleviate rheumatoid arthritis induced inflammation and suppress the growth of various tumors in mice (74, 75). Despite these encouraging findings, there might be a major limitation on the systematic administration of an inhibitor of glycolysis. Because the majority of somatic cells rely on glycolysis for energy, the risk for adverse events could be significantly high. However, in a phase I clinical trial (NCT02044861) evaluating the effect of PFK158, a PFK15-based synthetic compound, on patients with advanced solid malignancies, there were no severe side effects observed up to one-year post-treatment. This was also not observed in our study and previously *in vivo* studies (23, 76), thereby opening up possibilities to explore PFK15 as a potential therapy for IBD.

In summary, we found that PFKFB3 is highly expressed in stromal cells in IBD and that inhibition of PFKFB3 reduces inflammatory cytokine secretion by fibroblasts *in vitro* and attenuates *in vivo* experimental models of colitis. These data indicate the importance of PFKFB3 in the pathogenesis of IBD and open the possibility to evaluate PFKFB3-targeting clinical strategies.

## Data availability statement

The datasets presented in this study can be found in online repositories. The names of the repository/repositories and accession number(s) can be found in the article/**Supplementary Material**.

## Ethics statement

The studies involving human participants were reviewed and approved by Medical Ethics Committee of the Leiden University Medical Centre. The patients/participants provided their written informed consent to participate in this study.The animal study was reviewed and approved by The Dutch animal ethics committee and the Central Authority for Scientific Procedures on Animals (CCD).

## Author contributions

ZZ, LP, TP, EJ-M, and AE performed the experiments. ZZ, AM-J, BE, MB, and LH participated in the research design and interpretation of the data. ZZ, TP, MB, and LH contributed to the writing of the manuscript. All authors contributed to the article and approved the submitted version.

## Funding

This study was supported by the China Scholarship Council for providing Ph.D. fellowship grant 201806270259 (ZZ).

## Acknowledgments

We thank the Cell & Chemical Biology (CCB) of Leiden University Medical Center (LUMC) in Leiden, the Netherlands, for use of the slide scanner. We thank Stef Janson and Johan van der Reijden (Department of Gastroenterology and Hepatology, LUMC, the Netherlands), Dr. Lennard Voortman and Annelies Boonzaier-van der Laan (Dept. of CCB, LUMC, the Netherlands) for technical support. We thank the China Scholarship Council for providing ZZ a PhD fellowship. We also thank the clinicians from the Department of Gastroenterology and Hepatology, LUMC, for supplying patient samples.

## Conflict of interest

The authors declare that the research was conducted in the absence of any commercial or financial relationships that could be construed as a potential conflict of interest.

## Publisher’s note

All claims expressed in this article are solely those of the authors and do not necessarily represent those of their affiliated organizations, or those of the publisher, the editors and the reviewers. Any product that may be evaluated in this article, or claim that may be made by its manufacturer, is not guaranteed or endorsed by the publisher.
