# Supplementary material for: Increased stromal PFKFB3-mediated glycolysis in inflammatory bowel disease contributes to intestinal inflammation
Source: Front Immunol. 2022 Nov 2;13:966067. doi: 10.3389/fimmu.2022.966067 (PMC9670190; doi:10.3389/fimmu.2022.966067)
Supplement: Supplementary file 1 [file DataSheet_1.pdf]

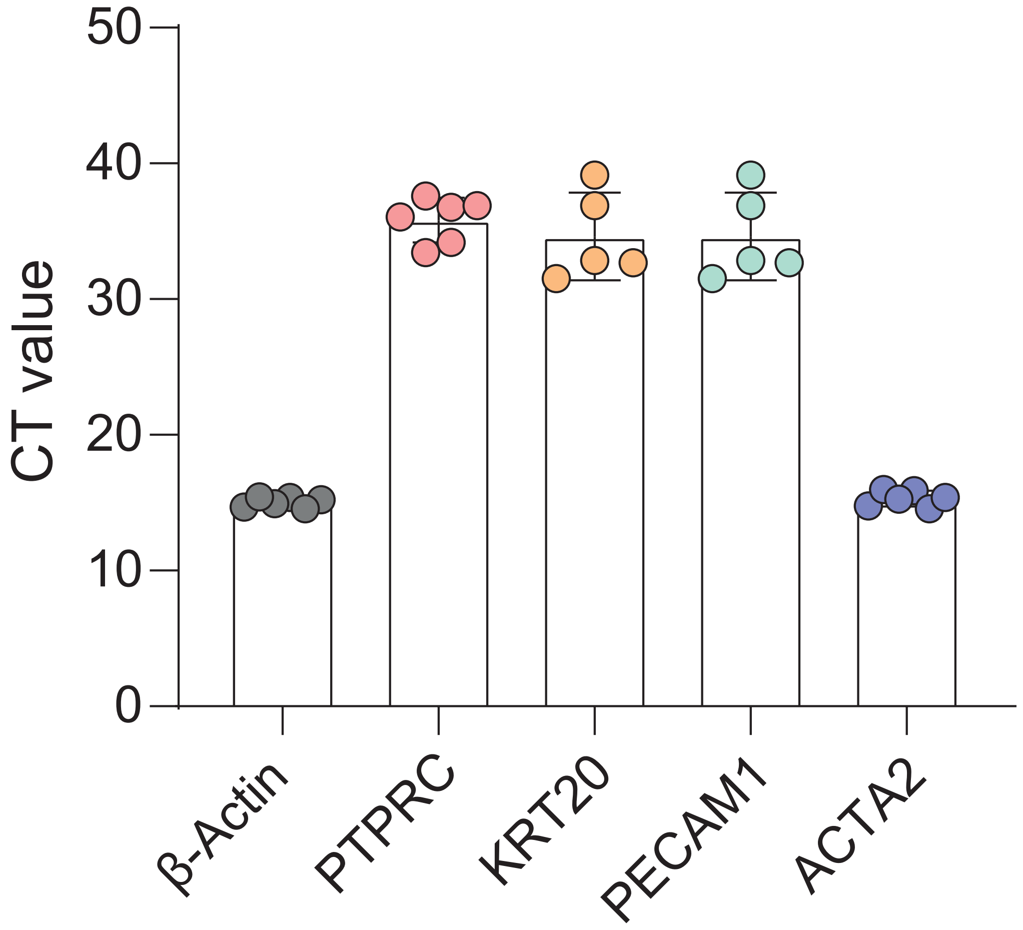

Supplementary Figure 1 Identification of fibroblasts. CT value of *β-Actin*, *PTPRC*, *KRT20*, *PECAM1*, and *ACTA2*.

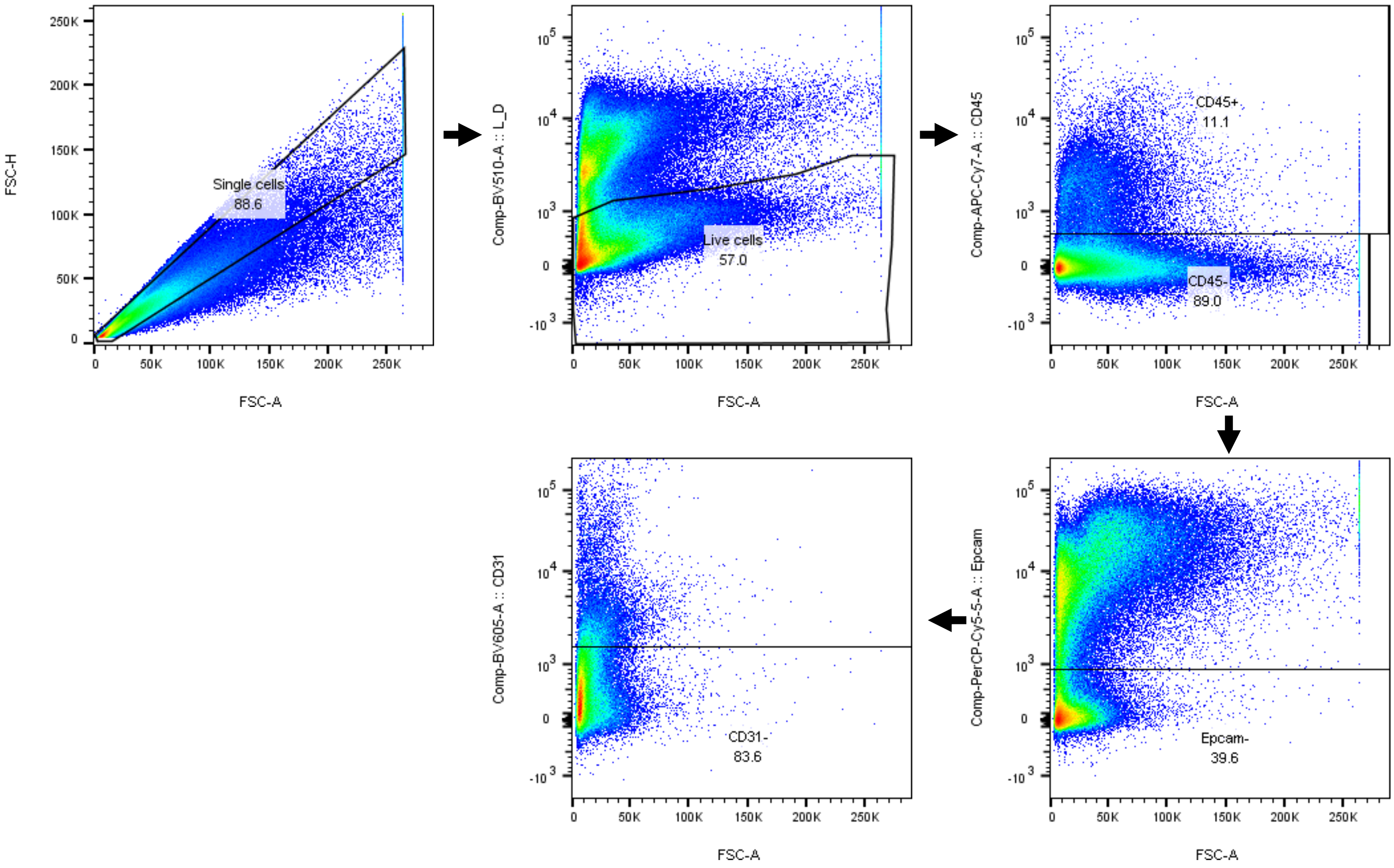

Supplementary Figure 2 Gate strategy of flow cytometry analysis. Dead cells, CD45+ cells, Epcam+ cells, and CD31+ cells were excluded.

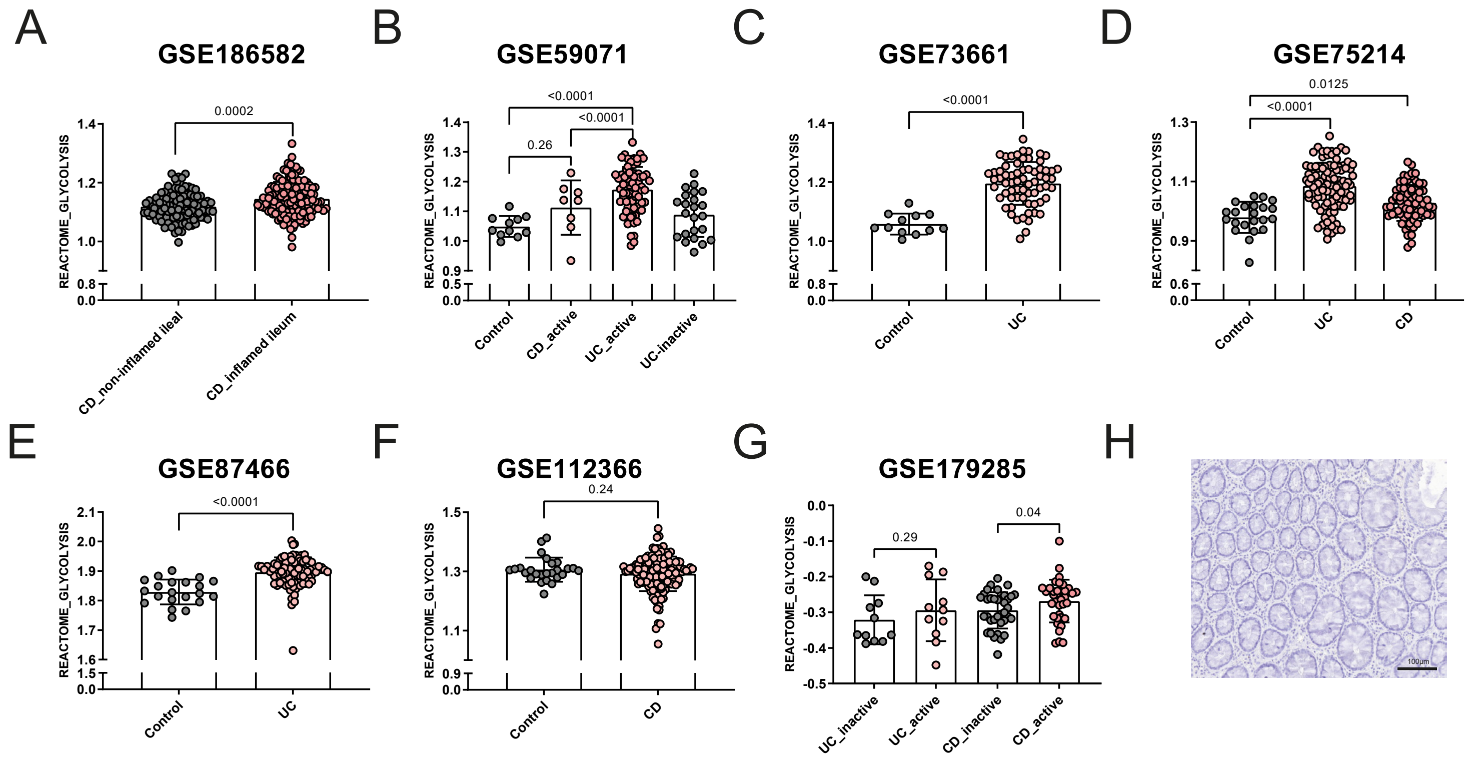

Supplementary Figure 3 Elevated glycolysis in IBD samples. ssGSEA analysis of GSE186582 **(A)**, GSE59071 **(B)**, GSE73661 **(C)**, GSE75214 **(D)**, GSE87466 **(E)**, GSE112366 **(F)**, and GSE179285 **(G)**, glycolysis score of each sample displayed in the graph. Non-paired/paired two-tailed t-test or one-way ANOVA were performed. **(H)** Staining control of PFKFB3, scale bar: 100μm.

A

Numbers of stromal cells in GSE114374

	PFKFB3 positive	PFKFB3 negative	Total	p value
Control	484 (0,15)	2800 (0,85)	3284 (1)	< 0,0001
UC	1151 (0,41)	1658 (0,59)	2809 (1)	

B

Numbers of stromal cells in GSE134809

	PFKFB3 positive	PFKFB3 negative	Total	p value
CD non-inflamed	480 (0,17)	2410 (0,83)	2890 (1)	< 0,0001
CD inflamed	720 (0,23)	2366 (0,77)	3086 (1)	

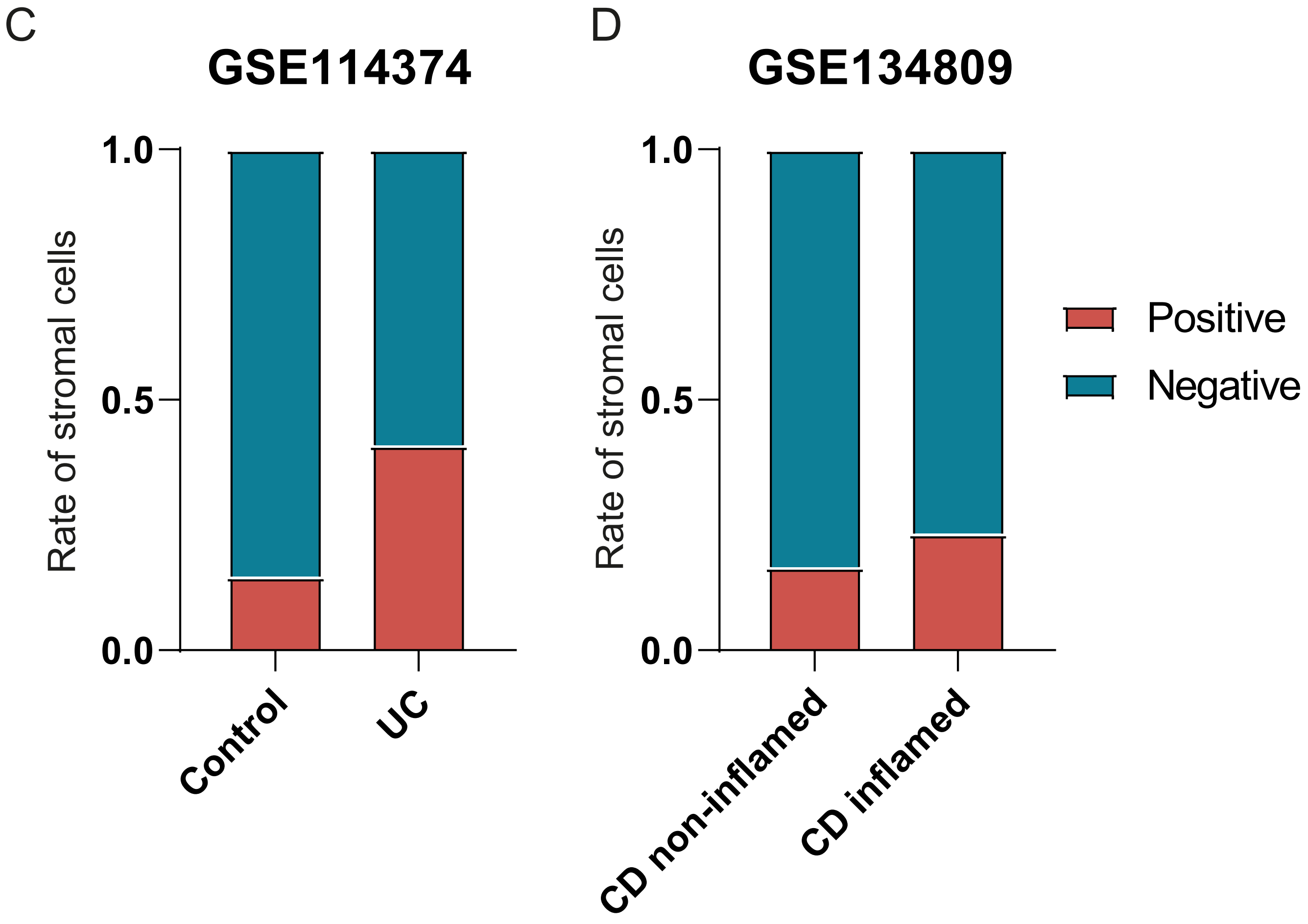

Supplementary Figure 4 Number and rate of PFKFB3 positive and negative stromal cells in GSE114374 and GSE134809. Fisher's exact test was performed to assess statical significance.

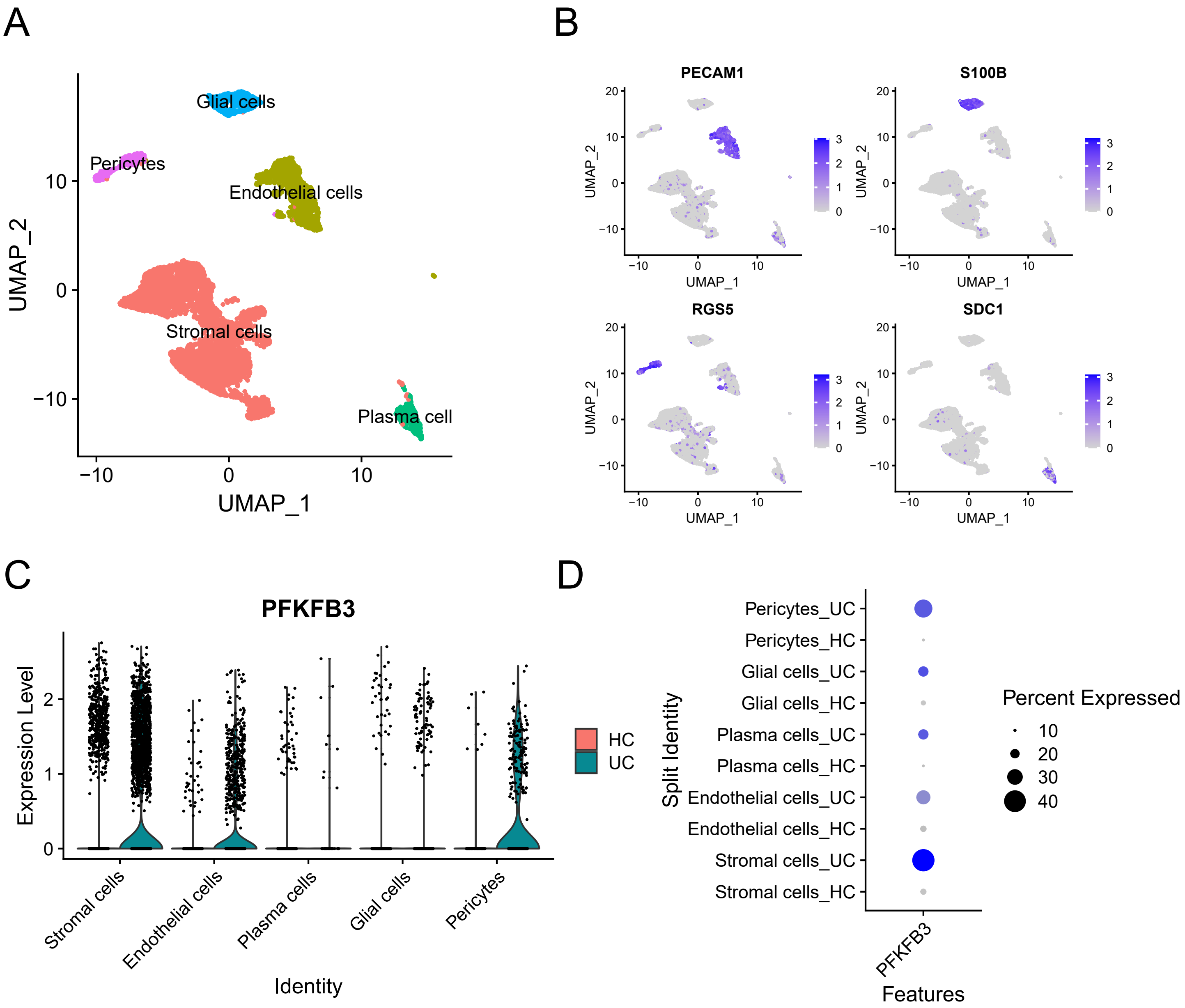

Supplementary Figure 5 PFKFB3 expression in different cell types in datasets GSE114374. **(A, B)** UMAP plot of stromal cells, endothelial cells, glial cells, pericytes, and plasma cells. PECAM1, S100B, RGS5, and SDC1 are the markers for endothelial cells, glial cells, pericytes, and plasma cells, respectively. **(C)** Violin plots of expression of PFKFB3 in each cell type of healthy control (HC) and UC samples. **(D)** Percentage of PFKFB3 positive cells in each cell type in HC and UC samples.

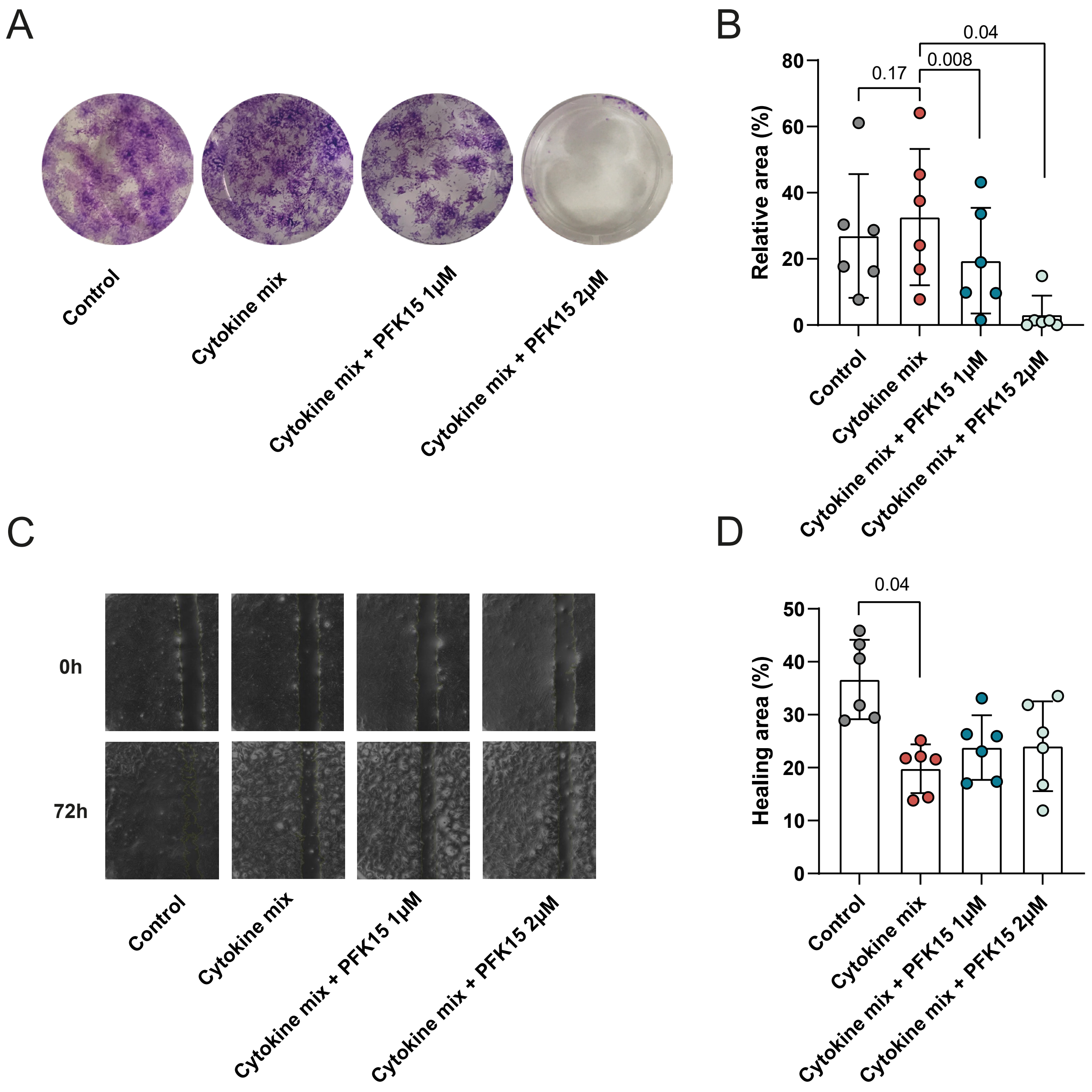

Supplementary Figure 6 PFKFB3 inhibition impaired the proliferation and migration of fibroblasts. Primary fibroblasts were treated with cytokine mix (IL17-A: 50ng/ml; OSM: 20ng/ml; IL-1β: 1ng/ml) and PFK15 (1μM or 2 μM) and the effects on proliferation and migration were studied. **(A)** Representative image from the colony formation assay, showing strongly decreased upon PFKFB3 inhibition. **(B)** Bar graph represented the statistical results of the relative colony area (n=6 independent fibroblast). **(C)** Representative phase-contrast microscope images showing the area covered by the cells at 0 and 72 h after wounding, showing decreased wound healing upon cytokine mix stimulation. **(D)** Quantification of the wound-healing assay. The data were normalized to the wound width of each group at 0 h (n=6 independent fibroblasts). One-way ANOVA was performed.

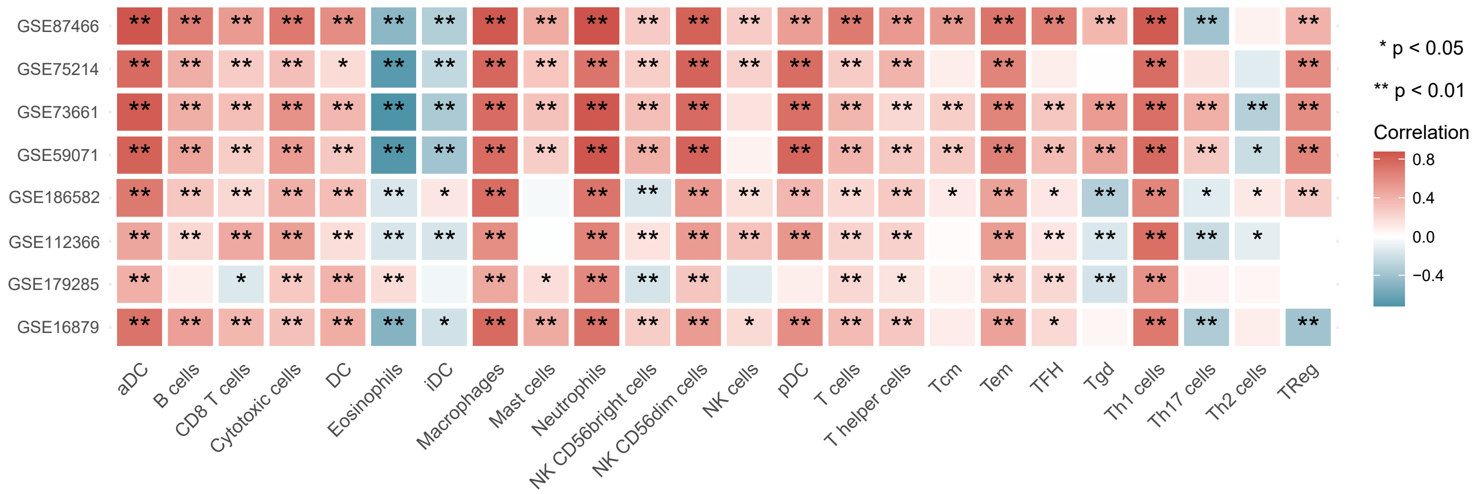

Supplementary Figure 7 Spearman's correlation analysis of PFKFB3 and immune cell infiltration in 8 datasets. Columns represent the relative amount of each immune cell, and rows represent datasets. Red represents positive correlation and blue represents the negative correlation of each immune cell. *p < 0,05, **p < 0,01. DC: dendritic cell, iDC: immature DC, aDC: activated DC, NK: natural killer cell, Tcm: T central memory, Tem: T effector memory, TFH: T follicular helper, Tgd: T gamma delta, Th: T helper, Treg: regulatory T cell.

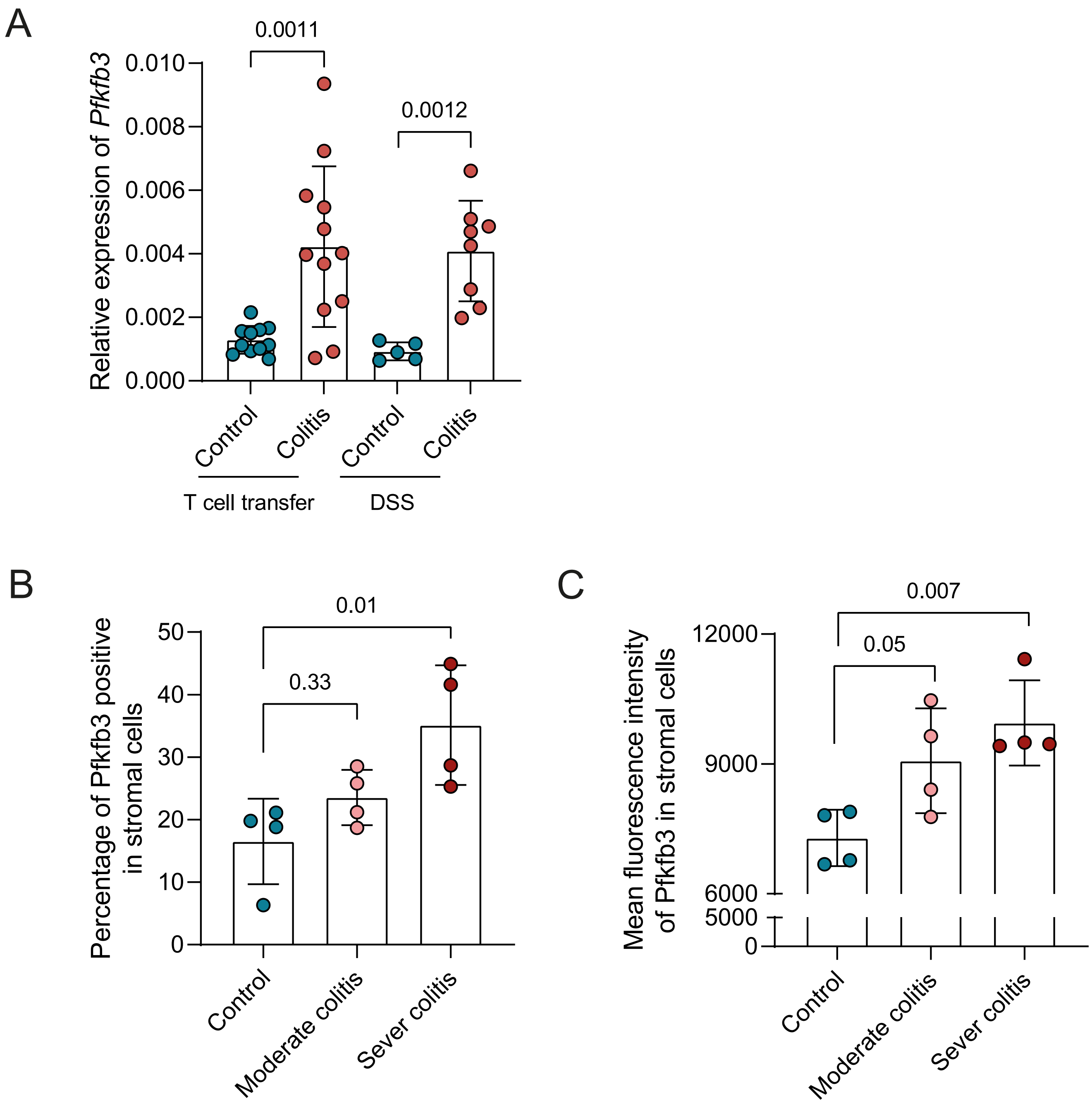

Supplementary Figure 8 Elevated Pfkfb3 expression in mouse colon samples. **(A)** Relative expression of Pfkfb3 corrected for β-actin as a housekeeping gene in T cell transfer colitis and DSS-induced colitis (T cell transfer colitis: control, n=11, colitis, n=12; DSS-induced colitis: control, n=5, colitis, n=8). **(B, C)** C57/BL6 mice were treated with DSS to induce different severity of colitis for 7 days (n=4/group). Colon was collected to perform flow cytometry. Dead cells, endothelial cells, immune cells, and epithelial cells were excluded. The percentage of Pfkfb3 positive stromal cells **(B)** and mean fluorescence intensity of Pfkfb3 in stromal cells **(C)** were displayed. The data are presented as the mean ± SD. Non-paired two-tailed t-test **(A)**, and One-way ANOVA **(B, C)** were performed.

Supplementary Table 1: Datasets of human IBD

	Platform	Samples
GSE16879	Affymetrix Human Genome U133 Plus 2.0 Array	61 IBD patients (24 UC, 19 Crohn's colitis (CDc), and 18 Crohn’s ileitis (CDi)), and 12 control patients (6 colon and 6 ileum)
GSE186582	Affymetrix Human Genome U133 Plus 2.0 Array	520 ileal biopsies from CD patients and 25 ileal non-IBD control
GSE59071	Affymetrix Human Gene 1.0 ST Array	97 UC, 8 CD, and 11 control colon samples
GSE73661	Affymetrix Human Gene 1.0 ST Array	67 UC patients and 12 non-IBD colonic biopsies
GSE75214	Affymetrix Human Gene 1.0 ST Array	97 UC (74 active, 23 inactive), 8 CDc (active), 22 control (11 colon, 11 ileum) and 67 CDi (51 active, 16 inactive)
GSE87466	Affymetrix HT HG-U133+ PM Array Plate	87 UC and 21 normal colon samples
GSE112366	Affymetrix HT HG-U133+ PM Array Plate	362 terminal ileum biopsies at baseline, 8 weeks after induction with Ustekinumab or placebo, and 44 weeks after maintenance from CD patients and 26 ileal controls
GSE179285	Agilent-014850 Whole Human Genome Microarray 4x44K G4112F	107 colonic and 70 ileal biopsies in the uninflamed area in all CD patients, additional inflamed regions of 35 colon and 55 ileum were obtained; paired uninflamed sigmoid (n = 48) and inflamed sigmoid biopsies were taken in UC patients; 58 control biopsies (21 sigmoid colon, 25 ascending/descending colon, and 12 terminal ileum)

Supplementary Table 2: The sequences of qPCR primers

Genes	Forward	Reverse
PFKFB3	CAGTTGTGGCCTCCAATATC	GGCTTCATAGCAACTGATCC
β-Actin	GTTGTCGACGACGAGCG	GCACAGAGCCTCGCCTT
CXCL9	CCAGGGGAGGTTCAGTGAAT	AGATAAGACGTTCGGGTGGG
CXCL12	CTCCACATCCTCCACGTTCT	GCTTTGGTCCTGAGAGTCCT
CXCL5	CAGACCACGCAAGGAGTTCATC	TTCCTTCCCGTTCTTCAGGGAG
IL-8	TCCTGATTTCTGCAGCTCTGT	AAATTTGGGGTGGAAAGGTT
TNF-α	TCTGGGCAGGTCTACTTTGG	TGAGCCAGAAGAGGTTGAGG
IL-1β	GAAGCTGATGGCCCTAAACA	AAGCCCTTGCTGTAGTGGTG
CD45	AACAGTGGAGAAAGGACACA	TGTGTCCAGAAAGGCAAAGC
CD31	GCTGACCCTTC TGCTCTGTT	TGAGAGGTGGTGCTGACATC
Keratin20	CAGACACACGGTGAACTATGG	GATCAGCTTCCACTGTTAGACG
α-SMA	CCGGGAGAAAATGACTCAAA	GAAGGAATAGCCACGCTCAG
Pfkfb3	GATCTGGGTGCCCGTCGATCACCG	CAGTTGAGGTAGCGAGTCAGCTTC
β-actin	AGGTCATCACTATTGGCAACGA	CCAAGAAGGAAGGCTGGAAAA
